# Prevalence of invasive fungal disease in hematological patients at a tertiary university hospital in Singapore

**DOI:** 10.1186/1756-0500-4-42

**Published:** 2011-02-28

**Authors:** Siok-Ying Lee, Chay-Leng Yeo, Winnie H Lee, Andrea L Kwa, Liang-Piu Koh, Li-Yang Hsu

**Affiliations:** 1 Pharmacy Department, Khoo Teck Puat Hospital, Yishun Central, Singapore; 2 Pharmacy Department, National University Hospital, Lower Kent Ridge Road, Singapore; 3 Pharmacy Department, Singapore General Hospital, Outram Road, Singapore; 4 Department of Hematology, Oncology, National University Hospital, Lower Kent Ridge Road, Singapore; 5 Department of Infectious Disease, National University Hospital, Lower Kent Ridge Road, Singapore

## Abstract

**Background:**

The use of newer azoles as prophylaxis in hematological patients undergoing stem cell transplantation or immunosuppressive chemotherapy has been shown to decrease the risk of developing invasive fungal disease (IFD). However, the cost-effectiveness of such a strategy is dependent on the local epidemiology of IFD. We conducted an audit of hematological patients with IFD in our institution in order to derive the prevalence and types of IFD that occur locally.

**Findings:**

We conducted a retrospective chart review of all hematological patients who developed possible, probable or definite IFD according to EORTC/MSG criteria in the period from Oct 2007 to Apr 2010. The prevalence of IFD was determined via correlation with institutional database records of all hematological patients treated at our institution over the same time period.

There were 39 cases of IFD diagnosed during the study period, with 8 (20.5%) possible, 19 (48.7%) probable and 12 (30.8%) definite cases of IFD. *Aspergillus *spp. accounted for 83.9% of all probable and definite infections. There was 1 case each of *Rhinocladelia *spp., *Coprinopsis cinerea*, *Exserohilum *spp. sinusitis and *Rhizopus *spp. sinusitis. IFD occurred in 12 of 124 (9.7%) AML and 4 of 103 (3.9%) ALL patients treated at our institution respectively. There were 10 (16.1%) infections among 62 allogeneic HSCT recipients, six of whom were having concurrent graft-versus-host disease (GVHD). Five other cases occurred after allogeneic HSCT failure, following salvage chemotherapy for disease relapse. The prevalence of IFD during induction chemotherapy was 8.9% (11 of 124 cases) for AML and 1.0% (1 of 103 cases) for ALL. Fluconazole prophylaxis had been provided for 28 out of the 39 (71.8%) cases, while 4 (10.3%) were on itraconazole prophylaxis. The in-hospital mortality was 28.2% (11 of 39 cases), of which 5 (12.8%) deaths were attributed to IFD.

**Conclusions:**

The burden of IFD is high in our institution, especially in allogeneic HSCT recipients and patients on induction chemotherapy for AML. A prophylactic strategy directed against invasive mould infections for local high-risk patients may be considered as the comparative costs of treatment, prolonged hospitalisation and subsequent delayed chemotherapy favours such an approach.

## Background

Antifungal prophylaxis is recommended for patients undergoing induction chemotherapy for acute myeloid leukemia (AML) and patients with allogeneic hematopoietic stem cell transplantation (HSCT) undergoing treatment for chronic extensive graft-versus-host disease (GVHD) [1,2]. It may also benefit other patient subpopulations at high risk of invasive fungal disease (IFD) - those with prolonged neutropenia after stem cell transplantation using bone marrow or cord blood sources, or those with impaired cell-mediated immunity secondary to therapeutic agents such as alemtuzumab [1]. Several key factors should be considered before deciding on whether to use prophylaxis and which agent to use. These include: the effectiveness against the types of fungi to be covered, institutional fungal epidemiology in the select group of patients with hematological malignancies, the number needed to treat, and the toxicity profile and drug interactions of the antifungal agent.

With the advent of new antifungals being used for prophylaxis for patients with hematological malignancies, fungal infection-related mortality has decreased [3]. However, this has also resulted in a shift in the epidemiology of invasive mycoses, with an increase in the prevalence of invasive mold infections among HSCT recipients and patients with hematological malignancies against a backdrop of a greater decline in prevalence of invasive candidiasis [4,5].

Currently at our hospital, fluconazole is the antifungal prophylactic agent used in patients with hematological malignancies receiving chemotherapy and allogeneic or autologous HSCT. Posaconazole, an extended-spectrum triazole, is active against most pathogenic yeasts and moulds [6], and has been approved for use as antifungal prophylaxis in the subgroup of allogeneic HSCT recipients with GVHD as well as profoundly neutropenic patients with AML and/or myelodysplastic syndromes (MDS) [6].

We performed an audit of hematology patients with IFD at our hospital - a 995-bed university hospital in Singapore with a large hematology and stem cell transplantation center. Our rationale for embarking on this study is to derive the epidemiology of IFD locally so as to determine if there is a role for a switch to posaconazole prophylaxis in the immediate future.

## Methods

A retrospective chart review was performed on all hematology patients diagnosed with IFD that were hospitalized between 1 October 2007 to 30 April 2010, outlining their outcomes and the prevalence of IFD according to the underlying hematological disease. All hematology patients with IFD in our institute are captured on a database at the point of diagnosis. In general, diagnostic workup for IFD was directed by patient symptoms, with the exception of persistent (>5 days) febrile neutropenia, where a computed tomography (CT) scan of the thorax was performed routinely. Serum galactomannan was performed weekly for HSCT recipients, while bronchoalveolar lavage (BAL) with galactomannan testing of BAL fluid was performed routinely for patients with CT thorax findings suggestive of fungal infection [7] but negative serum galactomannan. Tissue biopsies were performed where possible (platelet count >50,000 cells/μL).

The number of patients diagnosed with AML and acute lymphocytic leukemia (ALL) in the study period was derived from the hematology cytogenetics laboratory registry, while the number of allogeneic HSCT patients was obtained from the center's HSCT registry. IFD cases were classified as possible, probable or definite IFD according to the 2007 European Organization for Research and Treatment of Cancer Invasive Fungal Infections Cooperative Group and the National Institute of Allergy and Infectious Diseases Mycoses Study Group (EORTC/MSG) criteria, with physicians routinely screening through all CT pulmonary images if these had been performed looking for specific signs of fungal infection [7]. We followed EORTC/MSG recommendations for serum galactomannan optical density (OD) cutoffs, whereas the BAL galactomannan OD cutoff was >1.0 [8].

Variables collected included demographic and clinical data as well as outcomes - in-hospital mortality and IFD-related mortality. The latter was determined following chart review by an infectious diseases specialist, and was based on the presence of ongoing progressive fungal disease with no other apparent cause of death present.

This study is a subset of a larger febrile neutropenia program evaluation that was approved by the institutional ethics review committee (National Healthcare Group Domain Specific Review Board E/09/340).

## Results

During the study period, 103 and 124 patients were diagnosed and provided induction +/- consolidation therapy for ALL and AML respectively, while 135 patients received HSCT (62 allogeneic, including 6 cord blood transplants, and 73 autologous transplants) for a variety of haematological disorders. Locally, fluconazole capsules or less frequently itraconazole solution are routinely provided as antifungal prophylaxis to patients with leukemia receiving chemotherapy and HSCT recipients.

There were 39 cases of IFD diagnosed during this period, of which 8 (20.5%) were possible, 19 (48.7%) were probable and 12 (30.8%) were definite IFD according to EORTC/MSG criteria [7], with all definite IFD cases confirmed via histopathological detection of tissue-invasive fungi in biopsy specimens. 28 (71.7%) patients were on fluconazole, 4 (10.3%) were on itraconazole prophylaxis while the remainder received no antifungal prophylaxis. Demographic and clinical characteristics of these patients are shown in Table 1. The majority were male, and *Aspergillus *spp. accounted for 83.9% of all probable and definite infections. There was 1 case each of *Rhinocladelia *spp. and *Coprinopsis cinerea *pulmonary infection, 1 case of *Exserohilum *spp. pulmonary infection and sinusitis, and 1 case of *Rhizopus *spp. sinusitis.

**Table 1:** Demographic and clinical characteristics of hematology patients with invasive fungal disease.

Median age, years (range)	43 (17 - 88)
Male gender (%)	25 (64.1)

Hematological diagnosis:	
AML (% AML with IFD in study period)	12 (9.7)
ALL (% ALL with IFD in study period)	4 (3.8)
Autologous transplants (% autologous HSCT recipients with IFD)	1 (1.4)
Allogenic transplants (% allogeneic HSCT recipients with IFD)	10 (16.1)
- Neutropenia post-transplantation (% allogeneic HSCT recipients with IFD occurring during neutropenia)	4 (6.5)
- GVHD (% allogeneic HSCT recipients with IFD occurring during GVHD)	6 (9.7)
Relapsed leukemia on salvage chemotherapy post-transplantation	5
Others*	7

Location of IFD:	
Sinus (%)	5 (12.8)
Pulmonary (%)	34 (87.2)

Median serum galactomannan (range) for:	
- All IFD cases (*n *= 41)	0.3 (0.1--4.3)
- All probable/definite aspergillosis cases (*n *= 26)	0.6 (0.1--4.3)

*Others include 2 cases with MDS, 2 cases with aplastic anemia, and one case each of chronic myelomonocytic leukemia, angioimmunoblastic T-cell lymphoma, and hemophagocytic lymphohistiocytosis secondary to T-cell lymphoma.

The majority of cases of aspergillosis (96.5%) were identified by positive galactomannan testing from serum and/or BAL fluid. Fungal cultures from infected tissue or BAL samples were positive only in 17.9% of cases. *Rhinocladelia *spp. and *Coprinopsis cinerea *were identified by internal transcribed spacer (ITS) sequencing of infected lung tissue [9].

Eleven (28.2%) patients with IFD died during their hospitalization, with 5 (12.8%) deaths attributed to IFD. There was only non-aspergillosis mortality, who developed rapidly progressing mucormycosis involving ethmoid, maxillary, frontal and sphenoid sinuses as well as the left cavernous sinus. Extensive debridement and high-dose (10 mg/kg body weight) liposomal amphotericin B failed to control the infection and she died within one week of diagnosis. Intra-operative tissue specimens grew *Rhizopus *spp., while histology showed extensive disease - broad hyphal fungal fragments present in the blood vessels of the left inferior turbinate, within the lumina and invading the vessel walls and surrounding connective tissue stroma.

The prevalence of IFD in AML and ALL patients during induction chemotherapy was 8.9% (11 of 124 cases) and 1.0% (1 of 103 cases) respectively. The prevalence of IFD in allogeneic HSCT recipients was 16.1% (10 of 62 patients), occurring during initial neutropenia or GVHD.

## Discussion

Our audit showed that the prevalence of and mortality related to IFD is high in our institution, and comparable to previously published figures [4,5,10-13]. All cases of probable and definite IFD were caused by molds, and the paucity of invasive candidiasis in our patients can be attributed to the use of fluconazole or itraconazole as antifungal prophylaxis in hematological patients on chemotherapy or HSCT recipients. It is plausible that several possible IFD cases as defined by the EORTC/MSG criteria did not actually have fungal infection. However, it is not possible to differentiate these cases further given the current limitations of fungal diagnostics. The proportion of possible IFD relative to probable/definite IFD has decreased over time with more frequent use of BAL galactomannan testing [8].

Switching to posaconazole prophylaxis for at-risk patients may result in a significant reduction of IFD at our institution. Recent landmark trials have shown a relative risk reduction for IFD of 0.73 and 0.41 respectively during neutropenia and GVHD compared to fluconazole [10,11], with a number need to treat (NNT) of 16 and 27 respectively to prevent 1 fungal infection in each group [10]. To prevent 1 fungal infection-related death, the NNT was 27 and 33 for hematology patients with neutropenia and allogeneic HSCT recipients with GVHD respectively [14]. It is reasonable to assume that similar results can be expected locally, given comparable fungal epidemiology. However, our results also suggest that providing posaconazole for high-risk patients such as allogeneic HSCT recipients and AML patients on induction chemotherapy will cover just over 56% of potential future patients with IFD (assuming distribution of IFD among future local hematology patients remains constant), resulting in a reduction of approximately 42% of our current prevalence of IFD given expected success rates of posaconazole prophylaxis of 75%. These figures, while significant, suggest that more needs to be done to identify individual patients who are at risk for IFD.

The overall additional estimated cost of treating an IFD at our institution is approximately USD8,600.00 (data not shown), notwithstanding other adverse events such as a delay in chemotherapy and longer hospitalization. The current cost of posaconazole prophylaxis for a month is approximately USD2,300.00. Although several studies have shown that posaconazole prophylaxis is potentially cost-effective when compared to fluconazole [15-17], these are aggregate population-based results. The burden of payment for antifungal prophylaxis rests on the patients in our local setting as the cost of these drugs is borne directly by the patient or private insurance. Posaconazole prophylaxis on an individual level in Singapore may be viewed as being more of a hedge or insurance, and this may be a deterrent for those high-risk patients who lack the necessary funds to pay for the drug.

This study had several limitations. Firstly, this was a retrospective chart review and it is a plausible that even with the existence of database, not all cases of IFD were diagnosed. Secondly, there were no autopsies performed on the patients who had died. This may have led to an underestimation of the mortality that could be attributed to IFD.

## Conclusions

In conclusion, our audit revealed that the prevalence of IFD is high locally. Although posaconazole prophylaxis may potentially reduce these infections appreciably, a significant minority (>35%) of IFD occur in hematology patients where the data on the cost-effectiveness of posaconazole prophylaxis is lacking. More needs to be done to identify the patients and/or their risk factors in order to deliver the appropriate antifungal prophylaxis.

## Competing interests

LYH and AK have received research funding from Pfizer Inc. and Merck, Sharpe & Dohme. LPK is currently the site principal investigator for a multicenter study funded by Pfizer Inc. Other authors report no conflict of interest.

## Authors' contributions

SYL, CLY and LPK collected the data for the study. In addition, SYL wrote the first draft of the manuscript while LPK coordinated the study with LYH. WL and AK participated in the review of the manuscript. LYH conceived the study design and coordinated the study. All authors have read and approved the final manuscript.

## Authors' information

SYL is a pharmacist doing her residency in infectious disease at the Singapore General Hospital. CLY is a pharmacist involved in antimicrobial stewardship at National University Health System. WL and AK are principal clinical pharmacists at Singapore General Hospital who oversee the infectious disease residency program.

LPK is a consultant physician in the department of Hematology, Oncology at the National University Cancer Institute. LYH is an infectious disease physician at the National University Health System.
